# Accelerated Phenotypic Aging in Midlife is Associated with Long-term Risk of Neurodegeneration

**DOI:** 10.1093/geroni/igaf122.4057

**Published:** 2025-12-31

**Authors:** Huan Li, Mary Ryan Baumann, Jessica Malta, Lindsay Clark, Sterling Johnson, James Pankow, Art Walaszek, Natascha Merten

**Affiliations:** University of Wisconsin-Madison, Madison, Wisconsin, United States; University of Wisconsin-Madison, Madison, Wisconsin, United States; University of Wisconsin-Madison, Madison, Wisconsin, United States; University of Wisconsin-Madison, Madison, Wisconsin, United States; University of Wisconsin-Madison, Madison, Wisconsin, United States; University of Minnesota, Minneapolis, Minnesota, United States; University of Wisconsin-Madison, Madison, Wisconsin, United States; University of Wisconsin-Madison, Madison, Wisconsin, United States

## Abstract

PhenoAge is a multi-system aging measure based on easily-obtained clinical blood tests. It has been associated with increased risk of disability, age-related morbidities and mortality. Accelerated PhenoAge (PhenoAgeAccel) measures whether an individual is biologically/physiologically aging faster or slower than expected by their chronological age. It is unclear whether PhenoAgeAccel could also be a marker for increased risk of neurodegeneration. Neurofilament light chain (NfL) is a biomarker for general neurodegeneration, measurable in blood. In this study, we aimed to determine whether PhenoAgeAccel is associated with long-term changes in blood-based NfL levels from mid to later life. This longitudinal study is based on N = 1,508 (mean age=49 years, 54% women) Beaver Dam Offspring Study participants. We measured blood-based clinical markers necessary for calculating PhenoAgeAccel at baseline and serum NfL levels at baseline, 5-year and 10-year follow-up. We used linear mixed-effects models with PhenoAgeAccel as predictor and log NfL levels as outcome, adjusting for random intercept, study wave, sex, education, body mass index and kidney diseases. PhenoAgeAccel was not significantly associated with baseline NfL. With every additional year older in PhenoAge compared to chronological age (PhenoAgeAccel) at baseline, participants had faster NfL increases over 10 years [overall: 0.80% faster, 95% Confidence Interval(0.36%,1.25%); women:0.45%(-0.09%,1.00%); men:1.32%(0.57%,2.07%)]. Accelerated PhenoAge in midlife was associated with 10-year increases in NfL, overall and in men. Longer follow-up will be needed to determine whether PhenoAge is predictive of neurodegeneration later in life. PhenoAge could become a potential cost-effective measure to help identify individuals at risk of neurodegeneration and inform early intervention.

